# Tissue culture and generation of autotetraploid plants of **Sophora ﬂavescens** Aiton

**DOI:** 10.4103/0973-1296.71793

**Published:** 2010

**Authors:** Wei Kun-Hua, Gao Shan-Lin, Huang He-Ping

**Affiliations:** *Department of Genetics and Breeding, China Pharmaceutical University, Nanjing, Jiangsu - 211 198, People’s Republic of China*

**Keywords:** Chromosome determination, colchicines, micropropagation, **Sophora ﬂavescens** Aiton, tetraploid

## Abstract

**Background:**

**Sophora ﬂavescens** Aiton is an important medicinal plant in China. Early *in vitro* researches of **S. ﬂavescens** were focused on callus induction and cell suspension culture, only a few were concerned with *in vitro* multiplication.

**Objective:**

To establish and optimize the rapid propagation technology of **S. ﬂavescens** and to generate and characterize polyploid plants of **S. ﬂavescens**.

**Materials and Methods:**

The different concentrations of 6-benzylaminopurine (BAP), indole-3-acetic acid (IAA) and kinetin (KT) were used to establish and screen the optimal rapid propagation technology of **S. ﬂavescens** by orthogonal test; 0.2% colchicine solution was used to induce polyploid plants and the induced buds were identified by root-tip chromosome determination and stomatal apparatus observation.

**Results:**

A large number of buds could be induced directly from epicotyl and hypocotyl explants on the Murashige and Skoog medium (MS; 1962) supplemented with 1.4–1.6 mg/l 6-benzylaminopurine (BAP) and 0.3 mg/l indole-3-acetic acid (IAA). More than 50 lines of autotetraploid plants were obtained. The chromosome number of the autotetraploid plantlet was 2*n* = 4× = 36. All tetraploid plants showed typical polyploid characteristics.

**Conclusion:**

Obtained autotetraploid lines will be of important genetic and breeding value and can be used for further selection and plant breeding.

## INTRODUCTION

The dried root of **Sophora ﬂavescens** Aiton (Leguminosae), a typical traditional Chinese medicine, is commonly used for the treatment of viral hepatitis, cancer, viral myocarditis, gastrointestinal hemorrhage, and skin diseases (such as colpitis, psoriasis, and eczema). Early *in vitro* researches were focused on callus induction[1,2] and cell suspension culture.[3,4] Since 1989, the *in vitro* multiplication of **S. ﬂavescens** has been performed by using explants such as stem[5] and apical bud.[6] Up to now, there has been only one paper on the polyploid cell induction of **S. ﬂavescens** by colchicine treatment.[7] However, there has been still no report on the *in vitro* tetraploid induction of **S. ﬂavescens**. We sought a protocol for generating a tetraploid plant via the cluster buds from seed explants by colchicine treatment.

In plant breeding programs, polyploid plants have been used to develop superior varieties[8,9] and to restore the fertility of interspecific or intergeneric hybrids.[10,11] Furthermore, the leaves, stems, roots, and flowers in polyploid plants are usually bigger than those of the diploid plants. Thus, the polyploid plants may have an increased biomass and yield. The technique of *in vitro* polyploid induction with colchocine has been employed in many crops, such as tomato[12] and *Citrus sinensis*.[13] However, only a few of cases of the generation of polyploid medicinal plants have been reported to date. Compared with diploids, tetraploid plants of *Datura stramonium* have up to twice the alkaloid content in leaves, stems, and roots.[14]

The tissue culture-mediated induction of polyploid plants is advantageous, because when compared with traditional methods, tissue culture can obtain large number of materials for induction, and is more effective and convenient.[15] To develop superior varieties of **S. ﬂavescens**, we report here an *in vitro* protocol for multiplication and a method for generating polyploid plants by colchicine treatments. Autotetraploid lines of **S. ﬂavescens** were obtained and identified by root-tip chromosome determination and stomatal apparatus observation. These lines promise to be of important genetic and breeding value and will be used for further selection and plant breeding.

## MATERIALS AND METHODS

### Plant material

Seeds of **S. ﬂavescens** (2× = 18) were obtained from Lushi County, Henan Province, China. The original plant was identified by the Department of Genetics and Breeding of China Pharmaceutical University.

#### Seed disinfection and germination and culture conditions

Seeds of **S. ﬂavescens** (2× = 18) were sterilized by immersion in a 1% v/v sodium hypochlorite solution (containing three to five drops of Tween-20 per liter) for 8 min. The seeds were washed with sterile distilled water three to five times and then transferred to a Petri dish containing sterile filter paper to remove excess surface water. The surface-sterilized seeds were placed onto the Murashige and Skoog (MS; Murashige and Skoog 1962) medium containing 3% w/v sucrose at pH 5.8. The inoculated seeds were kept in an illuminated incubator for a 16-h photoperiod of 1200 lux light intensity at 25 ± 1°C.

#### Experiment on the bud proliferation medium by an orthogonal test

To increase the growth and quality of plantlets, an orthogonal test was used to select the best combination and concentration of phytohormones for inducing bud clusters. Three phytohormones, namely, 6-benzylaminopurine (BAP; 1.0, 2.0, and 3.0 mg/l), indole-3-acetic acid (IAA; 0.1, 0.2, and 0.3 mg/l), and kinetin (KT; 0, 0.5, 1.0 mg/l), were used at three concentrations each for the orthogonal test, and the MS medium was used as the basal medium throughout these studies. Fifty epicotyl or hypocotyl explants excised from seedlings were inoculated into 10 conical flasks for each of the 9 treatments defined above. The growth rate of buds [growth rate of buds = (harvested material weight – original material weight)/original material weight (g/g)] and multiplication time of buds [(harvested bud number – original bud number)/original bud number] were tested and evaluated 30 days after culture establishment. To obtain an objective evaluation about the effects of the bud proliferation medium, the configuration of buds and leaves was also observed as they developed.

#### Additional screening for bud proliferation

According to the results of the orthogonal test, the concentration of BAP was adjusted in a small range (1.0, 1.2, 1.4, 1.6, and 1.8 mg/l) to obtain an optimum rapid propagation medium for **S. ﬂavescens** with a fixed concentration of IAA (0.3 mg/l).

The sampled materials, culture conditions, and the parameters for evaluation were the same as in the previous test. After 30 days of culture, the effects on the buds were observed and recorded.

#### Induction of tetraploid plantlets

Fifty buds (approximately 2 cm in length) were excised from cluster buds. The buds were submerged in the 0.2% (w/v) colchicine solution for 0, 12, 24, 36, 48, 60, 72, 84, and 96 h, respectively. Treated buds were then transferred to the MS medium supplemented with 1.0 mg/l BAP and cultured in an illuminated incubator for a 16-h photoperiod of 1200 lux light intensity at 25°C for 20 days for bud proliferation. After that, all of the experimental materials were transferred to the MS solid medium supplemented with 1.5 mg/l BAP and 0.3 mg/l IAA for 30 days to induce cluster buds. The buds (approximately 3 cm in length) of the subculture materials were excised and transferred to the rooting medium consisting of the solid MS medium at half the macronutrient concentration and supplemented with 1.5 mg/l IBA to induce roots for subsequent chromosome determination.

#### Chromosome determination

Root tips approximately 0.5 cm in length were excised and pretreated in the 0.2% w/v colchicine solution for 3 h. After pretreatment, the root tips were transferred to Carnoy’s fixative (containing 3:1 ethanol and glacial acetic acid) and stored at 3–5°C for 2–24 h, rinsed with 95% (w/v) alcohol, 70% (w/v) alcohol, and distilled water, respectively, three times, and then macerated for 15 min with 0.2 M HCl at 60°C. After soaking in distilled water for 30 min, the fixed root tips were stained with improved Carbol fuchsin (1.8 g sorbitol dissolved in 10 ml Carbol fuchsin, and then mixed with 45% v/v acetic acid, 90 ml). A photomicroscope (Olympus BX40, Japan) was used for chromosome determination. The chromosome count of each tetraploid (4× = 36) line was repeated for at least three generations.

The buds (approximately 3 cm in length) of each tetraploid line were excised and transferred to the rooting medium consisting of the solid MS medium at half the macronutrient concentration and supplemented with 1.5 mg/l IBA to induce roots. And the rooted plants were transplanted into a seedling bed for leaf characteristics’ evaluation.

#### Estimation of leaf characteristics

Leaf characteristics were obtained from the 30-day-old *in vitro* material about 0.5 cm ^2^ in size and from 6-month-old fully established glasshouse plants 2–3 cm^2^ in size. For stomatal apparatus measurements, an area about 0.1 cm ^2^ on the upper epidermis of the unifoliate leaf was peeled off and spread onto a glass microscope slide. A photomicroscope (Olympus BX40) was used to measure the stomatal apparatus length and width. Four unifoliate leaves were chosen from the same part of each of five diploid control plants and each of five tetraploid plants. Twenty stomatal apparatus were measured for each leaf.

## RESULTS AND DISCUSSION

### Effects of phytohormones on multiplication

Growth regulators applied to plant culture media, such as auxins and cytokinins, are necessary for the accelerated micropropagation of different plant species. They can induce cell division, chloroplast development, shoot formation, and auxiliary bud outgrowth.[16] BAP is a one of the most important plant cytokinins. It can stimulate the following effects: cell division, lateral bud emergence, and basal shoot formation.[17] Kinetin (KT, N6-furfuryladenine) was the first cytokinin isolated and identified in 1955,[18,19] which can promote cell division in plants. IAA was the first plant hormone discovered by Went in 1928. Many studies have demonstrated that IAA plays a critical role in plant growth and development. IAA is thought to regulate or influence diverse responses on a whole-plant level, such as tropisms, apical dominance and root initiation, and responses on cellular level, such as cell enlargement, division, and differentiation.[20]

In our research, the orthogonal test revealed that the variation of the IAA concentration (4.18) had a more significant effect on the bud growth rate than other variables (variances ranged from 0.04 to 1.30) [Table 1]. Further optimization showed that the best growth rate was 6.12 g/g and was found at an IAA concentration of 0.3 mg/l [Table 2]. And the variation of the BAP concentration (3.30) had a more significant effect on the bud multiplication time than other variables (variances ranged from 0.04 to 0.67) [Table 3]. Future analysis showed that the best bud multiplication time was 9.09 and was found at a BAP concentration of 2.0 mg/l [Table 4]. The effect of KT on the bud multiplication time was not significant, which can be ignored. So from these results, we may draw a conclusion that the best multiplication medium for **S. ﬂavescens** was the MS medium supplemented with 2.0 mg/l BAP and 0.3 mg/l IAA. But with the inclusion of BAP in the medium at and above a concentration of 2.0 mg/l, abnormal growths such as fasciation and vitrification were observed. Bud clusters developed to produce normal and strong plantlets with green leaves if the BAP concentration was added to the medium at 1.0 mg/l. Considering the above situation, the MS medium containing 0.3 mg/l IAA and BAP at or below 2.0 had the best effects on plant propagation.

**Table 1:** Variance analysis of the bud growth rate of **S. ﬂavescens** on a propagation medium by an orthogonal test

Source of variance	Sum of variance squares	*df*	Variance	*F*-value	*P*-value
BAP	2.60	2	1.30	17.43*	0.05<P<0.1
IAA	8.36	2	4.18	55.91**	0.01<P<0.05
KT	0.08	2	0.04	0.53	>0.1
Error	0.22	2	0.11		
Sum	11.26	8			

F1 = 0.01(2, 2) = 99.0, F1 = 0.05 (2, 2) = 19.0, F1 = 0.1 (2, 2) = 9.0

* Significant at *P* = 0.1

** Significant at *P* = 0.05

**Table 2:** Visual analysis of the growth rate of *S. ﬂavescens in vitro* buds on the propagation medium by the orthogonal test

Concentration of phytohormone (mg/l)			Factor		
BAP	IAA	KT	A (BA)	B (IAA)	C (KT)
1.0	0.0	0.0	K_A_ 1/3 = 4.17	K_B_ 1/3 = 3.76	K_C_ 1/3 = 4.96
2.0	0.5	0.5	K_A_ 2/3 = 5.24	K_B_ 2/3 = 4.91	K_C_ 2/3 = 5.03
3.0	1.0	1.0	K_A_ 3/3 = 5.38	K_B_ 3/3 = 6.12	K_C_ 3/3 = 4.80
*R* (range)			1.20	2.36	0.22

The *K*-value is the sum of the growth rate of all tests with the same factor at the same level and the *R*-value is the difference between the maximum and minimum value of *K* with the same factor. The *K*-values and the effects of each level with the same factor are positive correlation; R-values and the effects of each factor are positive correlation

**Table 3:** Variance analysis of the bud multiplication time of **S. ﬂavescens** on the propagation medium by the orthogonal test

Source of variance	Sum of variance squares	*df*	Variance	*F* -value	*P*-value
BAP	6.60	2	3.30	19.93*	0.01<P<0.05
IAA	1.34	2	0.67	4.00	>0.1
KT	0.08	2	0.04	0.24	>0.1
Error	0.34	2	0.17		
Sum	8.36	8			

F1 = 0.01(2, 2) = 99.0, F1 = 0.05 (2, 2) = 19.0, F1 = 0.1 (2, 2) = 9.0

* Significant at *P* = 0.05

**Table 4:** Visual analysis of the bud multiplication time of *S. ﬂavescens in vitro* buds on the propagation medium by the orthogonal test

Concentration of phytohormone (mg/l)			Factor		
BAP	IAA	KT	A (BA)	B (IAA)	C (KT)
1.0	0.1	0.0	K_A_ 1/3 = 7.01	K_B_ 1/3 = 7.83	K_C_ 1/3 = 8.02
2.0	0.2	0.5	K_A_ 2/3 = 9.08	K_B_ 2/3 = 8.67	K_C_ 2/3 = 8.11
3.0	0.3	1.0	K_A_ 3/3 = 8.28	K_B_ 3/3 = 7.87	K^C^ 3/3 = 8.25
*R* (range)			2.08	0.83	0.27

The K-value is the sum of the bud multiplication time of all tests with the same factor at the same level and the R-value is the difference between the maximum and minimum value of K with the same factor. The K-values and the effects of each level with the same factor are positive correlation; R-values and the effects of each factor are positive correlation

**Figure 1:**
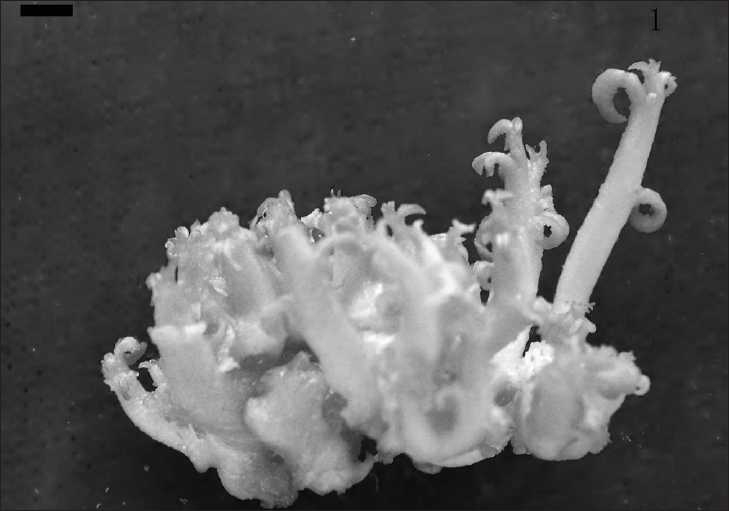
The buds of **Sophora ﬂavescens** Ait. (Leguminosae) on MS mediuma supplemented with 1.5 mg/l BAP and 0.3 mg/l IAA (bar: 0.154 cm)

Based upon the orthogonal test, further optimization experiments using BAP concentrations of 1.0, 1.2, 1.4, 1.6, and 1.8 mg/l were combined with a fixed IAA concentration of 0.3 mg/l. The fastest bud growth rate was 5.90 ± 0.14 g/g for cultures on a medium supplemented with BAP at 1.6 mg/l and the highest bud multiplication time was 9.88 ± 0.09 for cultures on a medium supplemented with BAP at 1.4 mg/l [Table 5; Figure 1].

Generally, the best multiplication medium for **S. ﬂavescens** was the MS medium supplemented with 1.4–1.6 mg/l BAP and 0.3 mg/l IAA.

**Table 5:** Effect of the BAP concentration on the bud growth of **S. ﬂavescens** when added to the MS medium supplemented with 0.3 mg/l IAA

Concentration of BAP (mg/l)	Average growth rate (g/g) x±SD	Bud multiplication time x±SD
1.0	4.31 ± 0.16	7.26 ± 0.12
1.2	4.54 ± 0.15	8.92 ± 0.13
1.4	5.13 ± 0.18	9.88 ± 0.09
1.6	5.90 ± 0.14	8.46 ± 0.13
1.8	5.33 ± 0.14	7.02 ± 0.16

### Effect of colchicine treatment on inducing buds with tetraploid buds

After treatment with 0.2% colchicine for increasing time, the percentages of death buds increased significantly, while all of the untreated buds survived. When the treated time was 12 h, the death rate of buds was 4.0%, and this response increased to 92.0% when the buds were exposed to 0.2% colchicine for 96 h [Table 6]. During the first culture stage of the tetraploid induction, the treated buds grew more slowly than the control when inoculated into the solid MS medium supplemented with 1.0 mg/l BAP for 20 days. But the treated buds were recovered after subcultured onto the MS solid medium supplemented with 1.5 mg/l BAP and 0.3 mg/l IAA for 30 days, and some were better than the control. This effect may be caused by the toxicity of the colchicine.[21] Colchicine is a phytoalkaloid that binds to tubulin and prevents its polymerization into microtubules, thereby blocking the formation of mitotic spindle and arresting nuclear division at metaphase.[22] Consequently, colchicine has long been used experimentally to visualize metaphase chromosomes in cytogenetic studies, and to induce polyploid in plants.[23] But the action of colchicine on the meristems may be cumulative and have a physiological disturbance resulting in a reduced rate of cell division or a death of explants.[24]

**Figure 2:**
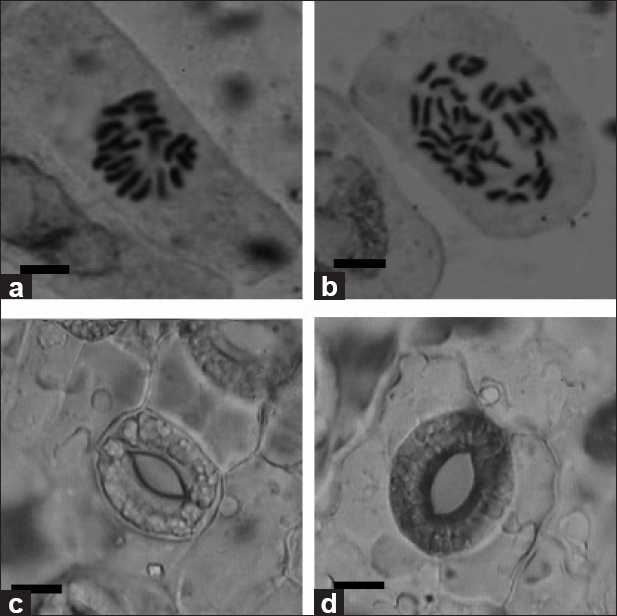
(a) The chromosome of diploid plant, 2*n*=2×=18 (bar: 6.04 × 10^-4^ cm). (b) The chromosome of tetraploid plant, 2n=4×=36 (bar: 6.04 × 10^-4^ cm). (c,d). Stomatal apparatus of diploid and tetraploid plants in **Sophora ﬂavescens** from glasshouse. Each Stomatal apparatus was obtained from the same part of diploid (c) and tetraploid (d) unifoliate leaves in the glasshouse (bar: 7.36 × 10^-3^ cm)

Chromosome determination was performed on root tips of the rooted plantlets. According to the chromosome counts, 51 plantlets were tetraploid. The data in Table 6 indicated that immersing buds in the 0.2% (w/v) colchicine solution for 12, 24, 36, 48, 60, 72, 84, and 96 h was efficient for the induction of buds to produce polyploid buds [Table 6]. The percentage of buds with polyploid buds was 28% when immersing buds in 0.2% colchicine for 48 h. This is by far the highest induction ratio in our experiments. Chromosome counts revealed that the tetraploid plantlets had 36 chromosomes (4× = 36) [Figures 2a and b].

**Table 6:** The effect of different treatments with the colchicine solution on polyploid induction in **S. ﬂavescens**

Number of treated buds	Duration of immersion (h)	Number of surviving buds	Death rate (%)	Number of tetraploids	Tetraploid rate (% of surviving buds)	Tetraploid rate (% of initial buds)
50	0	50	0.0	0	0.0	0.0
50	12	48	4.0	2	4.2	4.0
50	24	42	16.0	4	9.5	8.0
50	36	35	30.0	7	20.0	14.0
50	48	31	38.0	14	45.2	28.0
50	60	25	50.0	10	40.0	20.0
50	72	18	64.0	8	44.4	16.0
50	84	15	70.0	5	33.3	10.0
50	96	4	92.0	1	25.0	2.0

Treated buds were cultured for 20 days on a solid medium supplemented with 1.0 mg/l BAP for bud proliferation

#### Morphological differences between diploid and tetraploid

The morphological features of tetraploid plants were evaluated and compared with diploid control plants to determine whether they could be used to identify putative tetraploids. When compared with diploid plants, the leaves of tetraploid plants appeared normal in shape. The length, width, and number of the unifoliate leaves for 30-day-old diploid and tetraploid *in vitro* materials were not significantly different, but those of 6-month-old glasshouse-grown plants showed obvious difference. And these characteristics were significantly different when same materials from *in vitro* and glasshouse-grown leaves were compared [Table 7]. The length and width of *in vitro* diploid leaves were about 8.4 mm and 5.1 mm, respectively, while the same dimensions for *in vitro* tetraploid leaves were about 8.8 mm and 5.4 mm. The length and width of glasshouse-grown diploid leaves were about 30.3 mm and 14.0 mm, respectively, while the same dimensions in glasshouse-grown tetraploid leaves were about 36.0 mm and 17.9 mm, respectively. The unifoliate leaf numbers were both 3 for both 30-day-old diploid and tetraploid *in vitro* materials. However, the average unifoliate leaf number of 6-month-old glasshouse-grown diploid and tetraploid plants was 11.2 and 13.5, respectively. The surface area of glasshouse-grown tetraploid leaves was therefore about 2.04 times greater than that of leaves from diploid plants [Table 7].

**Table 7:** Leaf characteristics of diploid and tetraploid **S. ﬂavescens**

Characteristics	Diploid *(in vitro)*	Tetraploid *(in vitro)*	Diploid (glasshouse)	Tetraploid (glasshouse)
Unifoliate leaf length (mm)	8.4 ± 0.9^a^	8.8 ± 1.0^a^	30.3 ± 0.9^b^	36.0 ± 0.8^c^
Unifoliate leaf width (mm)	5.1 ± 0.5^a^	5.4 ± 0.5^a^	14.0 ± 0.9^b^	17.9 ± 1.0^c^
Unifoliate leaf area (cm^2^)	32.1 ± 2.7^a^	35.4 ± 2.5^a^	317.2 ± 4.7^b^	645.6 ± 6.8^c^
Average leaf number	3 ± 0^a^	3 ± 0^a^	11.2 ± 2.5^b^	13.5 ± 2.7^b^
Stomatal apparatus length (μm)	27.9 ± 1.2^a^	31.2 ± 1.9^b^	32.5 ± 1.9^b^	35.6 ± 1.0^c^
Stomatal apparatus width (μm)	24.3 ± 1.3^a^	25.1 ± 1.2^a^	27.7 ± 1.6^b^	30.5 ± 1.2^c^

Four leaves were chosen from each of five diploid control plants and each of five tetraploid plants. Twenty stomatal apparatus were measured for each leaf. Values represent the mean ± standard error. Within each row, means followed by the same letter are not significantly different at *P* = 0.05 level by Duncan’s multiple range test

The sizes of stomatal apparatus of *in vitro* diploid and tetraploid leaves as well as glasshouse-grown diploid and tetraploid leaves were measured and found to be significantly different [Table 7; Figure 2c and d]. In general, tetraploids possessed longer and wider stomatal apparatus. The length and width of the stomatal apparatus of *in vitro* diploid leaves were about 27.9 μm and 24.3 μm, respectively, while the same dimensions of *in vitro* tetraploid leaves were about 31.2 μm and 25.1 μm, respectively. The length and width of the stomatal apparatus of glasshouse-grown diploid leaves were about 32.5 μm and 27.7 μm, respectively, while the same dimensions in glasshouse-grown tetraploid leaves were about 35.6 μm and 30.5 μm, respectively. There was also a significant difference when the same ploidy material was grown under different conditions [Table 7]. The glasshouse-grown diploids material possessed longer and wider stomatal apparatus compared to *in vitro* diploids material. The same phenomena were found in tetraploid materials. Owing to the longer and wider stomatal apparatus of tetraploids, the utility of the stomatal size in distinguishing plants with different ploidy levels has been used in other plant types.[25] The stomatal apparatus of the *in vitro* material was smaller than that of the ex vitro material, which may be caused by the high humidity, weak light, and heterotrophic environment of the *in vitro* material.

Consequently, leaf sizes of glasshouse-grown plants and stomatal apparatus sizes of both *in vitro* and glasshouse-grown plant were useful parameters for identifying putative tetraploids in **S. ﬂavescens**. The increased size of roots, stems, leaves, and flowers are common characteristics of polyploid plants compared with diploid control plants. The larger stomatal apparatus of polyploid plants also could be considered to be a cytological screening factor for polyploid lines. And the bigger unifoliate leaf size of the glasshouse-grown tetraploids indicated that the higher biomass may yield greater amounts of the desirable compound.

In medicinal species, the leaves, stems, flowers, and roots are often the source of the desired active compounds, so the increased biomass associated with polyploid plants is a very attractive characteristic. The higher yield or higher active compound content of these plants is important for the extraction of natural products and their clinical use in many countries such as China and India. Matrine and oxymatrine are the chief active components in S. ﬂalescens. Matrine has a wide range of pharmacological actions, such as anti-inflammatory,[26] antidiarrhea,[27] analgesic,[28] antiarrhythmic,[29] antitumor,[30] and immunosuppressive effects.[31] Basic and clinical researches have shown that oxymatrine exhibits anti-inﬂammatory, immunosuppressive, antivirus,[32] liver-protective, and antihepatic ﬁbrosis activities.[33] In the present study, we have demonstrated the capacity to produce autotetraploid plants of **S. ﬂavescens**, an important step toward the goal of increasing production of matrine and oxymatrine.
